# Whole DNA methylome profiling in lung cancer cells before and after epithelial-to-mesenchymal transition

**DOI:** 10.1186/1746-1596-9-66

**Published:** 2014-03-20

**Authors:** Fatao Liu, Yi Zhou, Daizhan Zhou, Mengyuan Kan, Xiaomin Niu, Zhou Zhang, Di Zhang, Liming Tao, Lin He, Lixing Zhan, Yun Liu

**Affiliations:** 1 Institute for Nutritional Sciences, Shanghai Institute for Biological Sciences, Chinese Academy of Sciences, 320 Taiyuan RD, Shanghai 200031, PR China; 2 Bio-X Institute, Key Laboratory for the Genetics of Developmental and Neuropsychiatric Disorders (Ministry of Education), Shanghai Jiao Tong University, Shanghai 200030, PR China; 3 Department of Shanghai Lung Cancer Center, Shanghai Chest Hospital, Shanghai Jiao Tong University, Shanghai 200030, PR China; 4 Institute of Biomedical Sciences, Fudan University, Shanghai 200032, PR China; 5 Key Laboratory of Molecular Medicine, The Ministry of Education, Department of Biochemistry and Molecular Biology, Fudan University Shanghai Medical College, 138 YiXueYuan RD, Shanghai 200032, PR China

**Keywords:** DNA methylation, Epithelial-to-mesenchymal transition (EMT), Lung cancer, CpG, CGI, MSCC

## Abstract

**Background:**

Metastatic lung cancer is one of the leading causes of cancer death. In recent years, epithelial-to-mesenchymal transition (EMT) has been found to contribute to metastasis, as it enables migratory and invasive properties in cancer cells. Previous genome-wide studies found that DNA methylation was unchanged during EMT induced by TGF-β in AML12 cells. In this study, we aimed to discover EMT-related changes in DNA methylation in cancer cells, which are poorly understood.

**Methods:**

We employed a next-generation sequencing-based method, MSCC (methyl-sensitive cut counting), to investigate DNA methylation during EMT in the A549 lung cancer cell line.

**Results:**

We found that methylation levels were highly correlated to gene expression, histone modifications and small RNA expression. However, no differentially methylated regions (DMRs) were found in A549 cells treated with TGF-β for 4 h, 12 h, 24 h and 96 h. Additionally, CpG islands (CGIs) showed no overall change in methylation levels, and at the single-base level, almost all of the CpGs showed conservation of DNA methylation levels. Furthermore, we found that the expression of DNA methyltransferase 1, 3a, 3b (DNMT1, DNMT3a, DNMT3b) and ten-eleven translocation 1 (TET1) was altered after EMT. The level of several histone methylations was also changed.

**Conclusions:**

DNA methylation-related enzymes and histone methylation might have a role in TGF-β-induced EMT without affecting the whole DNA methylome in cancer cells. Our data provide new insights into the global methylation signature of lung cancer cells and the role of DNA methylation in EMT.

**Virtual slides:**

The virtual slides for this article can be found here: http://www.diagnosticpathology.diagnomx.eu/vs/1112892497119603

## Introduction

Lung cancer is one of the most common causes of cancer-related death [1]. In recent years, the expression and mutation of multiple genes have been shown to have a relationship with lung cancer [2-4]. Notably, metastasis and the related genetic and epigenetic changes are the main cause of the high death rate from lung cancer [5]. The migratory and invasive properties imparted by EMT (epithelial-to-mesenchymal transition) are known to be essential for metastasis [6]. EMT is characterized by the loss of cell-cell adhesion and other cell junctions, the loss of cell polarity, and the re-organization of the actin cytoskeleton. EMT is involved in metastasis in a variety of cancers, e.g., lung cancer [7], breast cancer [8] and prostate cancer [9]. Understanding the mechanisms of EMT may be a key approach for developing new therapeutic strategies for inhibiting tumor metastasis.

Methylation of DNA cytosine residues (5mC) has been proven to play important roles in many biological processes, such as embryonic development, organismal development and carcinogenesis [10]. Most studies have focused on 5mC in the CpG (C-phosphate-G) context [11]. Mammalian genomes are dominated by methylated CpGs, while there are also some hypomethylated domains, called CpG islands (CGIs), found at the 5′ region of genes, promoters and first exons [12]. CGIs are short CpG-rich regions with low methylation levels [13]. Many studies have shown that DNA methylation is associated with gene expression. Methylation of CGIs at transcription start sites (TSSs) is believed to contribute to gene silencing, and approximately 60% of human genes have CGI promoters [14], while methylation of CpGs in the gene body is positively correlated with gene expression [15], as it may correspond with elongation. Several histone modifications, such as H3K4me3, also have a role in the regulation of gene expression and cooperate with DNA methylation. Additionally, histone modifications help to direct DNA methylation patterning [16]. The ten-eleven translocation (TET) family of enzymes was recently found to catalyze the oxidation of 5mC to 5-hydroxymethylcytosine (5hmC), which might represent an intermediate form of an active DNA demethylation process and regulate gene expression [17].

The involvement of DNA methylation in EMT has long been considered. Hypermethylation in the promoter region was found to be one of the key factors that lead to reduced mRNA expression of E-cadherin during EMT [18]. Several genome-wide studies of global epigenetic changes during EMT have also been performed. Hundreds of genes showed EMT-associated changes in DNA methylation in EP156T cells, while there was no significant correlation between DNA methylation changes and changes in gene expression [19]. After induction with TGF-β, AML12 cells showed characteristics of EMT, but the genome-wide DNA methylation pattern was unchanged [20]. The relationship between global DNA methylation and EMT in cancer cells has not been revealed.

Here, to investigate possible EMT-related changes in DNA methylation in cancer cells, we employed MSCC (methyl-sensitive cut counting) to perform a genome-wide DNA methylation analysis in A549 lung cancer cells before and after EMT. We report the relationship between DNA methylation, histone modifications and gene expression in lung cancer cells, and we describe the weak effect of EMT on the whole DNA methylome. We also suggest that DNMTs (DNA methyltransferases), TETs and histone methylation patterns might have roles in TGF-β-induced EMT in cancer cells.

## Methods

### Cell culture and inducement of EMT

The A549 cell line was obtained from Mr. Ji Hongbin (Shanghai Institute for Biological Sciences, Chinese Academy of Sciences, Shanghai, China) and was maintained in RPMI 1640 medium supplemented with 10% FBS in a 37°C incubator with a humidified atmosphere of 5% CO_2_. TGF-β was purchased from R&D Systems (R&D Systems Inc., Minneapolis, MN, USA), and cells were treated at the time indicated with 5 ng/ml in culture medium to induce EMT. We repeated the experiments, including cell harvest followed by RT-PCR, Western blotting and DNA extraction, three times for each time point.

### Cell lysis and western blotting

Cells were rinsed with ice-cold PBS, were lysed in radioimmunoprecipitation assay (RIPA) buffer (CST, #9806) containing complete protease inhibitors (Roche), phosphatase inhibitors (Roche), 5 mM DTT (Sigma) and 1 mM PMSF, and were centrifuged at 15,000 x *g* for 10 minutes at 4°C. The supernatant was collected and then protein concentration was measured using the Bio-Rad protein assay (Bio-Rad). The proteins were separated by SDS-PAGE and blotted onto a PVDF membrane (Millipore). The antibodies used are listed in Additional file 1: Table S1.

### Isolation of RNA, reverse transcriptase-PCR, and quantitative real-time-PCR

Total RNA was isolated from A549 cells stimulated with TGF-β (5 ng/ml) for the indicated times using the RNeasy Mini Kit (Qiagen) according to the manufacturer’s instructions. One ug of RNA was reverse transcribed with the Takara PrimeScript RT Reagent Kit (Takara) following the manufacturer’s protocol to generate cDNA. The cDNA was used for real-time-PCR using the SYBR Premix Ex-Taq Kit (Takara) and was performed on a Bio-Rad CFX96 real-time PCR detection system (Bio-Rad). All expression data were normalized to β-actin expression levels. The primers used are listed in Additional file 1: Table S2.

### Genomic DNA extraction

For each time point of EMT, three dishes of cells were pooled for DNA extraction. Genomic DNA was extracted with the QIAamp DNA Mini Kit (Qiagen). DNA length was determined with an Agilent 2100 Bioanalyzer to ensure DNA integrity. Genomic DNA was then fragmented using a Gene Machine Hydroshear apparatus (Harvard Apparatus) at code 12 for 40 cycles. For each time point, we pooled the DNA together and performed MSCC library construction and sequencing to guarantee that the possible changes in DNA methylation induced by each time-dependent treatment could be detected.

### MSCC library construction and second-generation sequencing

The MSCC libraries were constructed according to the description of Guo et al. [21] with few alterations. For each of the five samples (S0h, S4h, S12h, S24h and S96h), two libraries were constructed. Two custom adaptors that contained a 5′ CG overhang and 3′ NN overhang, respectively, were created. For the *Hpa*II library, 2 μg of genomic DNA combined with standard DNA was digested with *Hpa*II (New England Biolabs [NEB]) for 2 h. Adaptor A was ligated to the resulting fragments. The reaction products were then incubated with *Bst* DNA polymerase (NEB) for 20 min. After digestion with *Mme*I (NEB), adaptor B was added to the reaction mixture incubated with T4 DNA ligase (NEB) overnight. The products were purified with Agencount AMPure XP Beads (Beckman) and then run on a 2% E-Gel® EX Gel (Invitrogen). The target band at approximately 140 bp was purified with the QIAquick Gel Extraction Kit (Qiagen). An 8-cycle PCR protocol was performed on the purification products. For the inverse library, after *Hpa*II digestion in the first step, the fragmented ends were deactivated by incubation with Antarctic Phosphatase (NEB). The products were digested with *Msp*I and then treated with the same procedure as the *Hpa*II library.

### MSCC sequencing and data analysis

First, the MSCC libraries were pooled. A total of two lanes of sequencing was performed on an Illumina HiSeq2000 sequencing system. The sequencing reads contain an 18-bp ‘tag’ and part of the sequence of Adaptor A. The index sequences within Adaptor A were used to distinguish different MSCC libraries (Additional file 1: Table S3). For each library, after adaptor removal, the 18-bp tags were mapped to the dataset of all possible 18-bp tags in the human genome (hg19) using MOM software [22]. Reads were accepted if they were uniquely mapped to the dataset with less than 2 mismatches. For each CCGG site, sequencing reads from the *Hpa*II library came from the CCGG sequence, and those from the inverse library came from the C^m^CGG or C^hm^CGG sequence. The data are summarized in Additional file 1: Table S4. After normalizing the number of reads in both libraries by the counts of standard DNA, the methylation level of each site was calculated. The sequenced sites were then mapped to known genes and CGIs using relevant tables downloaded from the UCSC genome browser (http://genome.ucsc.edu/).

### Analysis of gene expression data

The gene expression data for the A549 cell line was obtained from GEO DataSets (http://www.ncbi.nlm.nih.gov/geo/, (GEO accession GSE17708)). The expression data of more than 20,000 genes was used for our analysis. For each gene, the average of the expression data resulting from all corresponding probes was defined as the expression level. The expression data at the 0 h (0 hour) time point was used for analysis in our study.

### Histone modification and short RNA sequencing data

The histone modification and short RNA sequencing data were downloaded from GEO DataSets (GEO accession GSE35583 and GSM897083). The broad peaks of histone modifications and the locations of short RNAs were extracted and analyzed.

### Statistical analysis

We used 200-bp non-overlapping windows and known CGIs as units to compare genome-wide DNA methylation. For each of these units (>3 sequenced CpGs), the average methylation level of the mapped CpGs was calculated. A p value was also assigned using Fisher’s exact test by comparing each unit in different samples.

## Results

### Morphology of the A549 cell line and characterization of EMT

**Figure 1:**
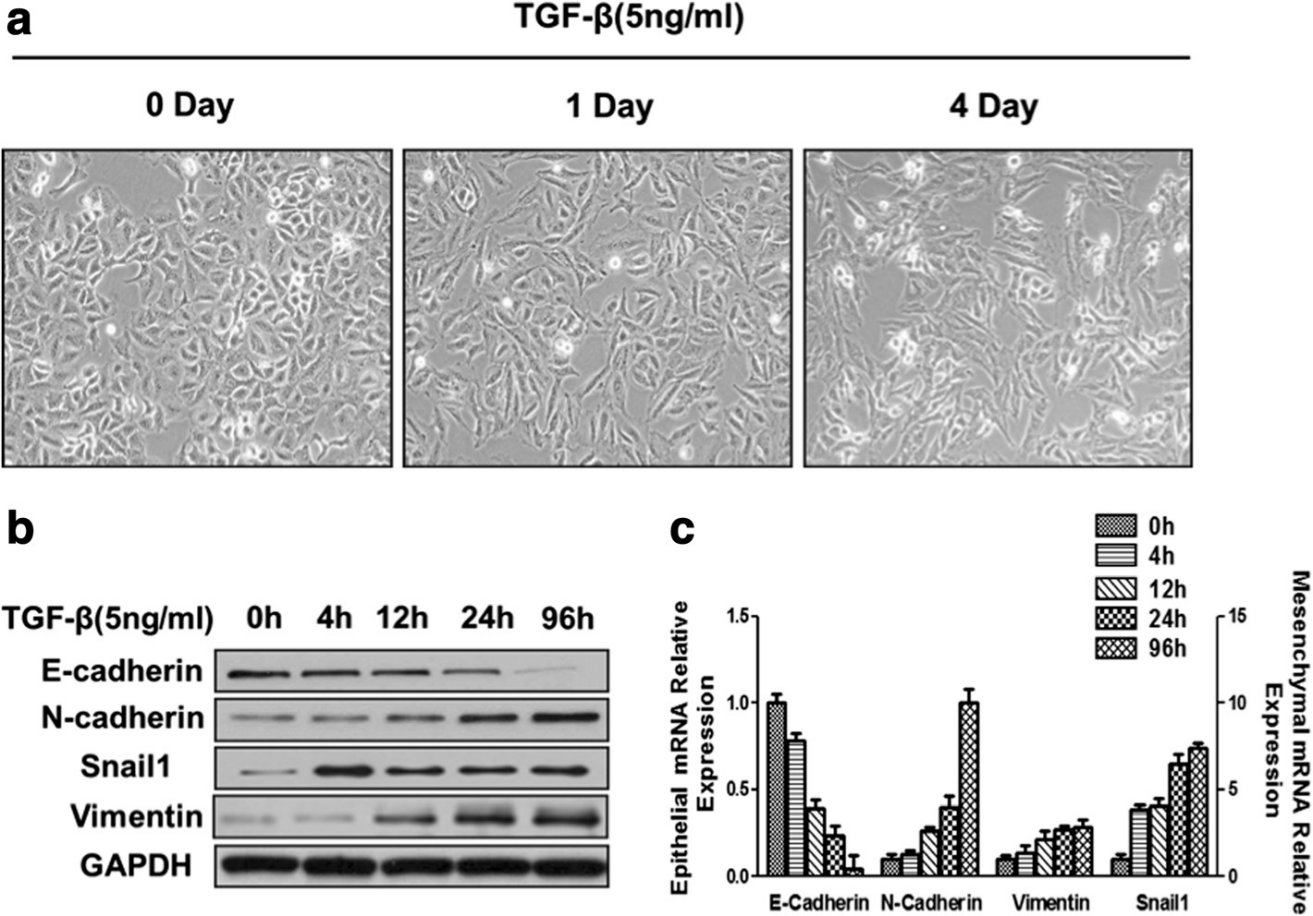
**Characteristics of the A549 cell line before and after EMT. (a)** The morphology of A549 cells treated with TGF-β (5 ng/ml) for different time intervals was visualized by phase-contrast microscopy (Olympus). **(b)** The expression of E-cadherin, N-cadherin, Vimentin, and Snail in TGF-β-treated A549 cells was analyzed by western blotting. **(c)** mRNA extracted from A549 cells treated with TGF-β was analyzed by RT-PCR (E-cadherin is shown on the left *y*-axis, and N-cadherin, Vimentin, and Snail1 are shown on the right *y*-axis).

A549 human lung cancer cells exhibited a pebble-like shape. After induction with TGF-β for 1 day and 4 days, the A549 cells gradually changed from an epithelial to elongated mesenchymal phenotype (Figure 1a). The phenotypic change was accompanied by a progressive loss of epithelial markers and gain of mesenchymal markers. At both the protein and mRNA levels (Figure 1b,c), the expression of E-cadherin was downregulated and the expression of N-cadherin, Snail1 and Vimentin was upregulated. After treating A549 cells with TGF-β for 4d, we found that the cells were substantially elongated and the expression of E-cadherin had reduced to near-zero levels (Figure 1a,b,c). Thus, we chose the 4 d time point for our post-EMT sample and analyzed a total of 5 time points (S0h, S4h, S12h, S24h and S96h) in the following study.

### Global properties of DNA methylation in the A549 cell line

**Figure 2:**
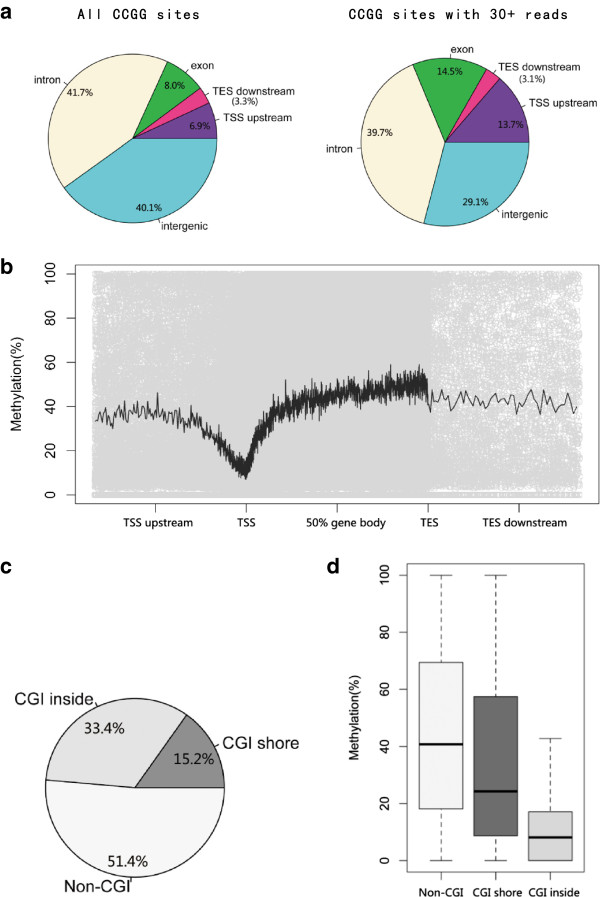
**Global properties of DNA methylation in the A549 cell line. (a)** The distribution of all CCGG sites and those with more than 30 reads. CCGG sites with 30+ reads are similarly distributed with all CCGG sites. **(b)** The distribution of CCGG methylation levels from the perspective of genes (hollow dots). The black line shows the moving average methylation level. **(c)** The CCGG sites with 30+ reads were mapped to CGIs and CGI shores. More than half of the CCGG sites are located in CGIs or CGI shores. **(d)** Overall methylation levels of the CCGG sites located in the respective regions.

The sequencing depth of each CCGG site significantly impacts the estimation of DNA methylation levels (Additional file 1: Figure S1), as Guo et al. [21] has described. To ensure accuracy and adequate CG coverage in all 5 samples, we chose CCGG sites with more than 30 sequencing reads for the following analysis. The selected sites were mapped to known human genes. The majority of the sites are located in introns and intergenic regions (Figure 2a). Additionally, due to the randomness of second-generation sequencing, the distribution of CCGG sites with 30+ reads was very similar to that of all the CCGG sites in the human genome. Furthermore, according to the data of Guo et al., the distribution of CCGG sites corresponded to all CG sites; thus, the methylation of sites with 30+ reads may well reflect the genome-wide methylation status.

From chromosomal analysis, we found that most chromosomes had similar overall methylation levels, while the Y chromosome and the mitochondrial genome were hypermethylated (Additional file 1: Figure S2). The calculated methylation levels of CCGG sites were plotted based on the relative positions of the nearby genes (Figure 2b). The moving average is also shown. Most of the positions had similar average methylation levels except for the regions surrounding the TSS (transcription start site), which were poorly methylated. The analyzed sites were also mapped to known CGIs and CGI shores. Approximately one half of the sites were located in CGIs or CGI shores (Figure 2c). As expected, the CCGG sites within CGIs showed significantly lower overall methylation levels compared to those located outside of CGIs (Figure 2d). In addition, CGI shores showed intermediate methylation levels.

### The methylation pattern of A549 cells is closely related to gene expression, histone modification status and short RNA coding regions

**Figure 3:**
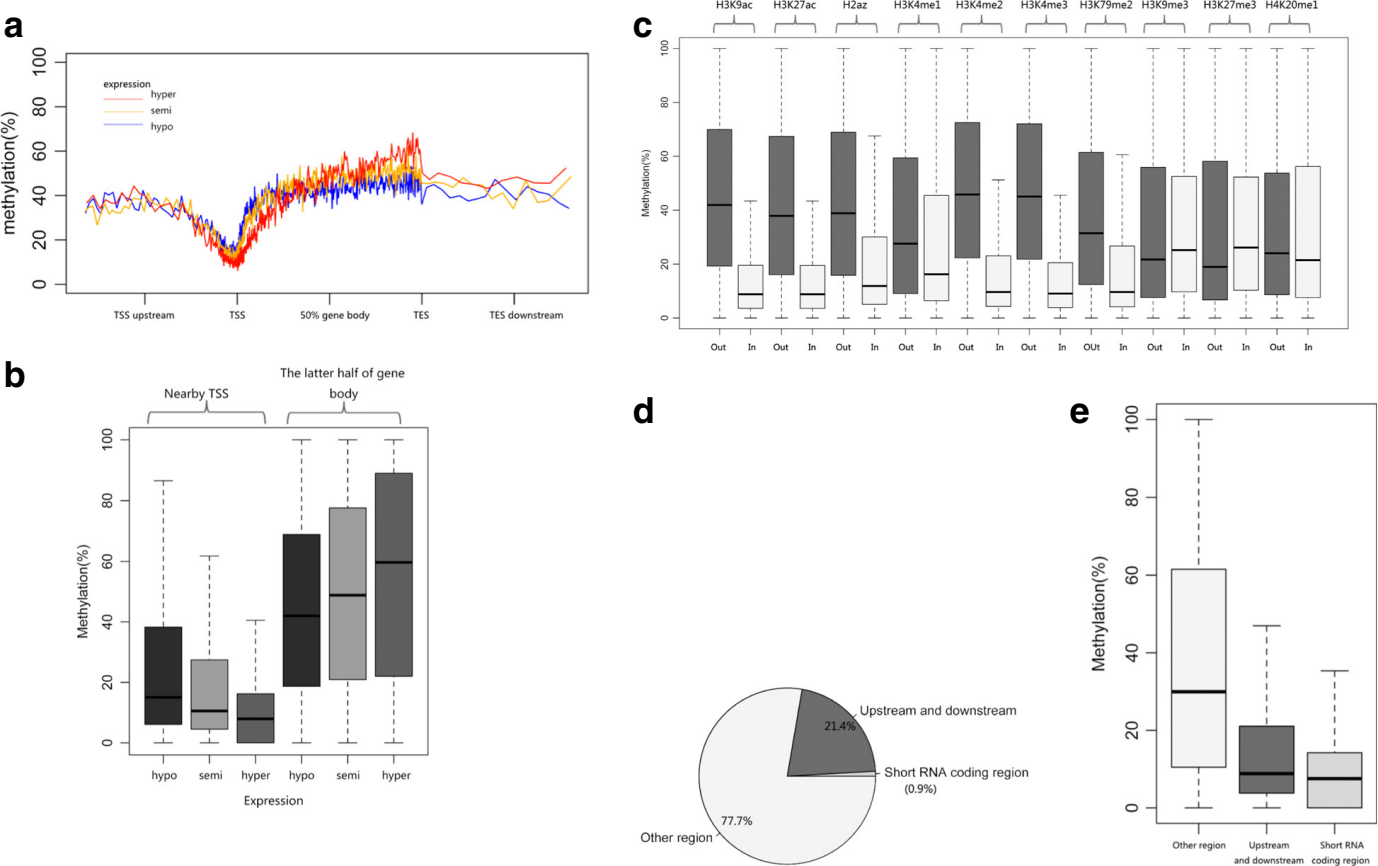
**DNA methylation levels closely correlate with gene expression, histone modification and short RNA expression. (a)** The methylation level of genes with high (red line), medium (orange line) and low (blue line) expression levels. **(b)** The TSS region and latter half of the gene body show differential DNA methylation levels that correspond to gene expression level. **(c)** Comparison of DNA methylation levels within and without differentially histone bound regions. The histone modifications analyzed include histone acetylation and methylation. **(d)** The percentage of CCGG sites located in and near short RNA coding regions. **(e)** The overall methylation level of CCGG sites that are within, near and without short RNA coding regions.

First, we studied the effect of DNA methylation levels in different regions of genes on gene expression. The expression data of more than 20,000 genes of the A549 cell line were used. The genes were divided into three groups in accordance with gene expression level. The methylation levels of genes with hypo-, semi- and hyperexpression levels were analyzed. As shown in Figure 3a, the three groups of genes exhibited similar DNA methylation distribution; however, there were significant differences between the three groups in DNA methylation in TSS regions and gene bodies. Genes with high expression levels had the lowest methylation levels near the TSS and highest methylation levels in the latter half of the gene body (Figure 3a and b). The situation for genes with low expression levels was just the opposite. The distribution of methylation and expression level of genes may influence each other. The top 20 genes with the highest and lowest average methylation levels around the TSS region are listed in Additional file 1: Table S5.

We then considered the relationship between histone modifications and DNA methylation. The CCGG sites were mapped according to relative location to the peaks of two types of histone acetylation and eight types of histone methylation. The overall methylation level of sites within and without the peaks of each type of histone modification was compared. We found that histone acetylation, including H3K9ac and H3K27ac, has significant influence on DNA methylation levels. CCGG sites within histone acetylation peaks had lower levels of methylation compared to those without the peaks (Figure 3c). There was also a close relationship between histone methylation and DNA methylation. Peaks of histone methylation, including H2az, H3K4me1, H3K4me2, H3K4me3 and H3K79me2, showed lower DNA methylation levels than the regions of the genome lacking these histone modifications. However, peaks of H3K9me3 and H3K27me3 had slightly higher methylation levels. H4K20me1 also had a very weak effect on DNA methylation (Figure 3c). It is likely that histone modifications may serve as marks of DNA methylation.

Short total RNA (RNAs shorter than 200 nucleotides) sequencing data were also analyzed. Only 0.9% of CCGG sites were located in short RNA coding regions, while a large percentage (21.4%) were within 2000 bp upstream or downstream of the coding regions (Figure 3d). We noticed that short RNA coding regions and nearby regions had lower DNA methylation levels than the other regions of the genome (Figure 3e). From our data, short RNA expression may be influenced by DNA methylation occurring via specific mechanisms.

### After EMT, the whole DNA methylome was unchanged

**Figure 4:**
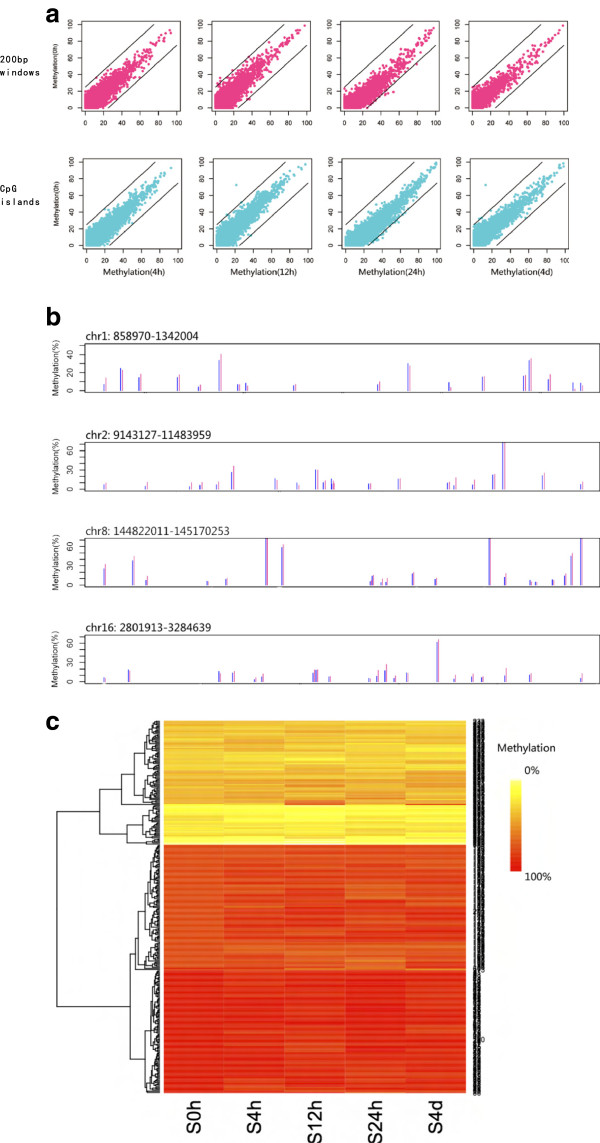
**Comparison of genome-wide DNA methylation of A549 cells before and after EMT. (a)** The average methylation level of 200-bp windows (violet red dots) and CGIs (cyan dots) of S4h, S12h, S24h and S96h compared with S0h. A 25% difference boundary line is shown (black line). **(b)** Example regions to show that the average methylation level of the 200 bp windows is similar in S0h (blue line) and S24h (violet red line). **(c)** The average methylation level of CGIs that were hypo-, semi- and hypermethylated in primary A549 cells. The data of 100 CGIs are shown as examples.

Because adjacent CG sites always have similar DNA methylation levels and to avoid the discovery of false positives, we first scanned the genome using 200-bp consecutive non-overlapping windows to find differentially methylated regions. For each 200-bp window (> 3 CpGs involved), the average methylation level was compared between A549 cells before and after EMT. A p value was also assigned using Fisher exact tests. Using a cutoff of 25%, few 200-bp windows with changes in methylation were found in the S4h, S12h, S24h and S96h treatment groups among all of the > 10,000 200-bp windows analyzed (Figure 4a). The average methylation levels of 200-bp windows from several randomly selected genomic regions in the S24h compared with S0h groups are shown in Figure 4b. After treatment with TGF-β, the whole-genome methylation status of A549 cells was unchanged.

We also analyzed the effect of TGF-β-induced EMT on the methylation status of CGIs in the genome of A549 cells. The average methylation level of more than 10,000 CGIs was calculated and compared between S4h, S12h, S24h, S96h and S0h. We found that the genome-wide DNA methylation pattern was unchanged. No CGI consistently showed a >25% change in methylation after the cells were treated with TGF-β for different times (Figure 4a). The methylation levels of 100 CGIs that had the highest, lowest and intermediate methylation levels in primary A549 cells are shown in Figure 4c. From the above data, TGF-β-induced EMT has no effect on the methylation status of genomic CGIs.

Changes in the whole methylome were also considered at the single-base level. The methylation levels of all the CCGG sites in S4h, S12h, S24h and S96h were closely correlated with those in S0h (Additional file 1: Figure S3). Additionally, among the approximately 160,000 CCGGs quantified in S0h, S4h, S12h, S24h and S96h (Additional file 2: Table S6), almost no CpGs (<0.1%) had a consistent > 25% methylation change in the treated samples. Approximately all of the CpGs showed conservation of methylation levels.

### EMT affected histone methylation and expression of methylation-related enzymes

**Figure 5:**
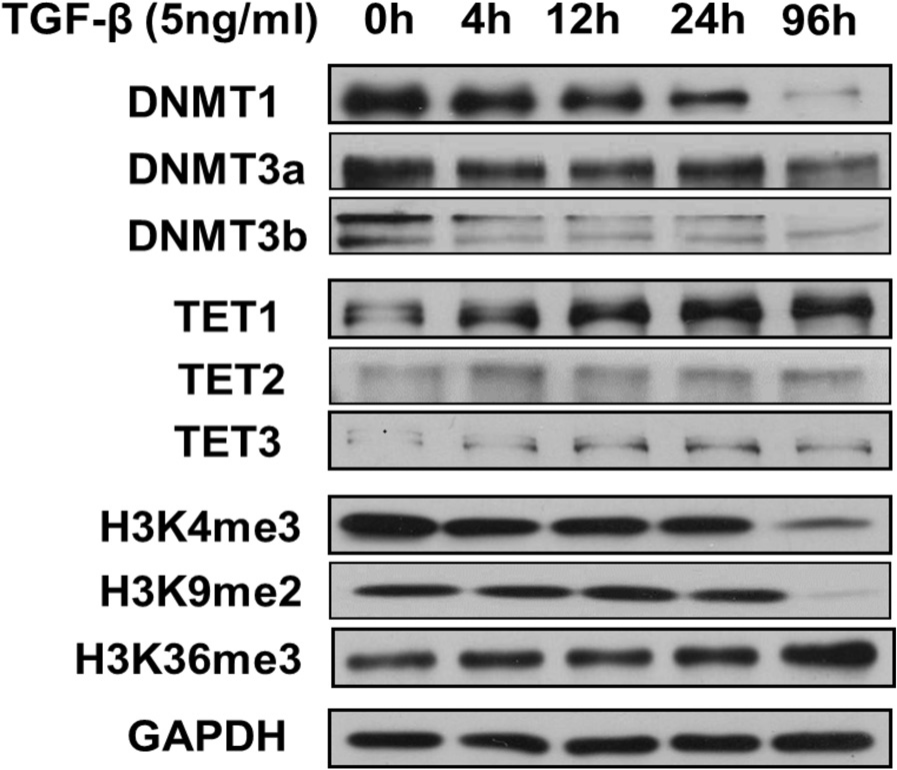
**Expression of methylation-related enzymes and histone methylation levels in A549 cells before and after EMT.** Changes in DNMTs, TETs and several types of histone methylation after TGF-β treatment over different time intervals were examined by Western blotting.

We then studied whether TGF-β-induced EMT influences histone methylation levels and the expression of methylation-related enzymes at the protein level. We found that DNMT1, DNMT3a and DNMT3b were significantly downregulated, and TET1 was significantly upregulated. Low expression levels of TET2 and TET3 were observed and conserved during EMT. We also noticed that the level of H3K4me3 and H3K9me2 histone methylation in A549 cells was significantly reduced after co-stimulation with TGF-β for 96 h. However, the level of H3K36me3 was increased in S96h (Figure 5).

## Discussion

Lung cancer metastases are common in people living with lung cancer and significantly contribute to lung cancer death [5]. EMT is believed to play crucial roles in cancer metastasis [6]. In the present study, we analyzed a genome-wide DNA methylation profile and studied the relationship between TGF-β-induced EMT and the whole DNA methylome in lung cancer cells. We report the global methylation signature of A549 cells and its relationship with gene expression, histone modifications and short RNA coding regions. Furthermore, our data provide evidence that TGF-β-induced EMT had almost no effect on the global DNA methylome. Notably, we found that expression of DNMTs and TETs were altered after EMT. Additionally, histone methylation modifications including H3K4me3, H3K9me2 and H3K36me3, which were demonstrated to have a role in the EMT of AML12 cells [20], were also changed during cancer cell EMT in our study.

TGF-β-induced rapid senescence of A549 cells has long been noticed [23-25]. Detection of cell markers including E-cadherin, N-cadherin, Snail1 and Vimentin has been used to identify typical EMT processes [26]. TGF-β induces changes in these cell markers and cell shape by activating diverse downstream pathways [27-29]. According to our data, after TGF-β treatment, A549 cells showed characteristics of EMT.

From the methylation signature of the A549 cell line, we observed low methylation levels at the TSS region of the genes. This may be due to the lack of nucleosomes, which are required substrates of de novo methylation, at active TSSs [11,30]. A low overall methylation level of CGIs in A549 cells was also found. CGIs are protected from methylation through two possible mechanisms: recognition of common *cis*-acting sequences [31,32] and active demethylation [33]. The relationship between DNA methylation and gene expression has long been considered. Methylation in the immediate vicinity of the TSS blocks the initiation of gene expression, and high gene expression levels may also in turn block de novo methylation of the promoter [34]. The genes with the lowest average methylation levels around the TSS included FANCD2, FAM58A, HDAC1, and C14orf166 according to our data, and FCGBP, CAPN12, FES, and CBLC had the highest average TSS methylation levels in A549 cells. Higher methylation within the gene body of genes with hyperexpression was also found in the present study. It has been proposed that transcriptional elongation may stimulate DNA methylation and that H3K36me3, which is also associated with elongation, might be involved in the recruitment of DNMTs [35].

The methylation signature of the genome is also closely correlated with many histone modifications, and we observed an effect of histone acetylation and methylation on CCGG methylation. It has been found that acetylated histones are associated with open chromatin, and unmethylated DNA tends to get repackaged into an open configuration. Conversely, non-acetylated histones are often associated with compact chromatin and methylated DNA [12,36,37]. Different types of histone methylation also serve as marks for the genome-wide DNA methylation pattern. An inverse relationship between H3K4me and H2az histone marks and DNA methylation has long been considered [30,38], and here, we report that H3K79me2 is anti-correlated with CCGG methylation. Additionally, H3K9me3 and H3K27me3 have been found to be repressive marks of gene transcription and positively correlated with DNA methylation by interacting with methylated DNA-binding proteins [39-41]. Furthermore, our data indicate that DNA methylation might be associated with short RNA expression.

In A549 cells undergoing EMT, we found that there was no genome-wide DNA methylation reprogramming, and we observed changes in H3K4me3, H3K9me2 and H3K26me3. The regulation of gene expression by histone modifications and DNA methylation has long been considered [42-45]. Histone modifications provide unstable transcriptional repression, and DNA methylation serves as a highly stable silencing mark that is not easily reversed [16]. During just 4 days of A549 cell treatment with TGF-β, the status of histone modifications was easily changed. However, DNA methylation remains relatively stable and cannot be altered in a short time, while the expression of DNMTs and TETs was regulated according to our data. The altered expression levels of the genes during and after EMT that were found by Sartor et al. [46] may not have been induced by altered DNA methylation but by altered histone modifications.

In the present study, we quantified the methylation levels of approximately 300,000 CCGG sites, which is approximately 13% of all CCGG sites and 1.1% of all CG sites in humans; thus, the methylation status of other CG sites and non-CG sites were not included. Although the methylation levels of the CCGG sites sequenced in our study could well reflect the methylation status of all CG sites, the employment of some higher throughput DNA methylation methods, for example, reduced representation bisulfite sequencing (RRBS) and whole-genome bisulfite sequencing (WGBS), may further confirm our findings. We tested for changes in whole histone methylation levels during EMT, and ChIP-Seq technology will help generate high-resolution profiles of histone modifications and allow more detailed study of the role of histone modifications in EMT of cancer cells. Additionally, more detailed molecular biology research may provide information regarding the direct role of DNMTs and TETs in EMT. In summary, our work explored the genome-wide DNA methylation signature of A549 lung cancer cells and the influence of EMT on the epigenome. Although the exact role of DNMTs, TETs and histone modifications in TGF-β-induced EMT and whether the levels of these influence genome-wide DNA methylation over longer periods of time were not determined, our findings may contribute to the uncovering of the epigenetic mechanisms of EMT in cancer cells and benefit the development of cancer metastasis treatments.

## Conclusions

We investigated the whole DNA methylome of cancer cells during EMT for the first time, and we found that DNA methylation-related enzymes and histone methylation might have a role in TGF-β-induced EMT without affecting the whole DNA methylome of cancer cells. Our data provide new insights into the global methylation signature of lung cancer cells and the role of DNA methylation in EMT and may benefit the development of cancer metastasis treatments.

## Competing interests

We declare that we have no competing interests.

## Authors’ contributions

FTL, YZ, GYF, LH, LXZ and YL designed the research; FTL, YZ, DZZ, MYK, XMN, ZZ, DZ and LMT conducted the research; FTL and YZ wrote the paper; FTL had primary responsibility for the final content. All authors read and approved the final manuscript.

## Supplementary Material

Additional file 1: Figure S1 The effect of sequencing depth on the accuracy of methylation level estimation. For each level of sequencing depth, the methylation of all the CCGG sites involved was showed (S0h on x-axis and S24h on y-axis). As the sequencing depth increases the Pair-wise correlation coefficient increases, which indicates that the sequencing data is more believable. **Figure S2.** The overall methylation of each chromosome of primary A549 cells. The mitochondrial genome and Y chromosome show significantly high methylation level. The average methylation level of other chromosomes is approximately 20%. **Figure S3.** The methylation level of CCGG sites with 30+ reads in S4h, S12h, S24h and S4d compared with S0h. The Pair-wise correlation coefficients are showed and a similarity between cells before and after EMT is observed. **Table S1.** List of antibodies used for western blot. **Table S2.** List of primers used for qRT-PCR. **Table S3.** List of Index used in second-generation sequencing. **Table S4.** Summary of second-generation sequencing data in MSCC-seq. **Table S5.** The top 20 genes with the highest and lowest average methylation level around TSS region. Click here for file

Additional file 2: Table S6 The methylation level and annotation information of CCGGs quantified both in S0h, S4h, S12h, S24h and S4d. Click here for file
