# Supplementary material for: Whole DNA methylome profiling in lung cancer cells before and after epithelial-to-mesenchymal transition
Source: Diagn Pathol. 2014 Mar 20;9:66. doi: 10.1186/1746-1596-9-66 (PMC4108085; doi:10.1186/1746-1596-9-66)
Supplement: Additional file 1: Figure S1 — The effect of sequencing depth on the accuracy of methylation level estimation. For each level of sequencing depth, the methylation of all the CCGG sites involved was showed (S0h on x-axis and S24h on y-axis). As the sequencing depth increases the Pair-wise correlation coefficient increases, which indicates that the sequencing data is more believable. Figure S2. The overall methylation of each chromosome of primary A549 cells. The mitochondrial genome and Y chromosome show significantly high methylation level. The average methylation level of other chromosomes is approximately 20%. Figure S3. The methylation level of CCGG sites with 30+ reads in S4h, S12h, S24h and S4d compared with S0h. The Pair-wise correlation coefficients are showed and a similarity between cells before and after EMT is observed. Table S1. List of antibodies used for western blot. Table S2. List of primers used for qRT-PCR. Table S3. List of Index used in second-generation sequencing. Table S4. Summary of second-generation sequencing data in MSCC-seq. Table S5. The top 20 genes with the highest and lowest average methylation level around TSS region. [file 1746-1596-9-66-S1.docx]

**SUPPLEMENTARY INFORMATION**

**Supplementary Figure S1**

**
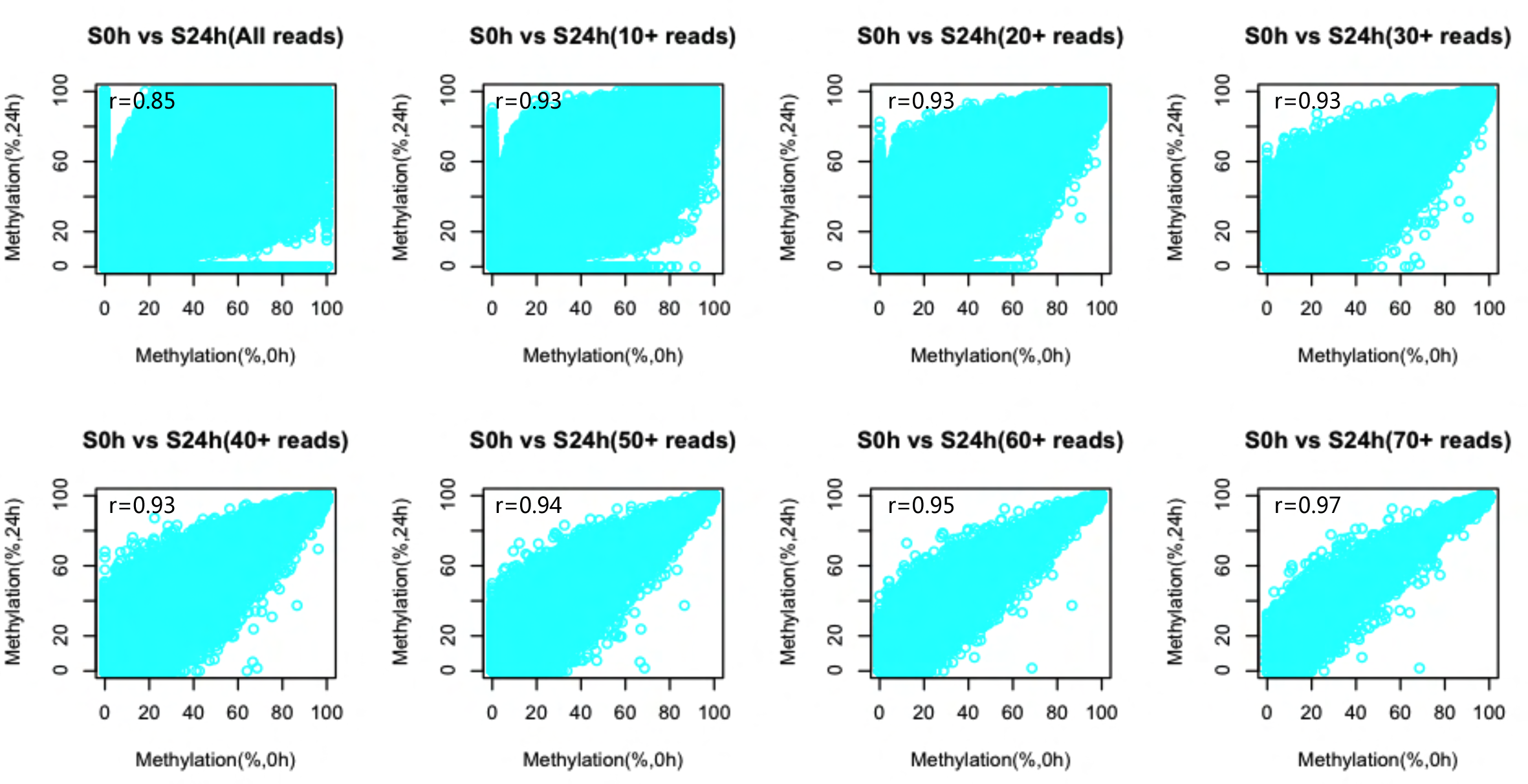
**

**Supplementary Figure S1:** The effect of sequencing depth on the accuracy of methylation levelestimation. For each level of sequencing depth, the methylation of all the CCGG sites involved was showed (S0h on x-axis and S24h on y-axis). As the sequencing depth increasesthe Pair-wise correlation coefficient increases, which indicates that the sequencing data is more believable.

**Supplementary Figure S2**

**
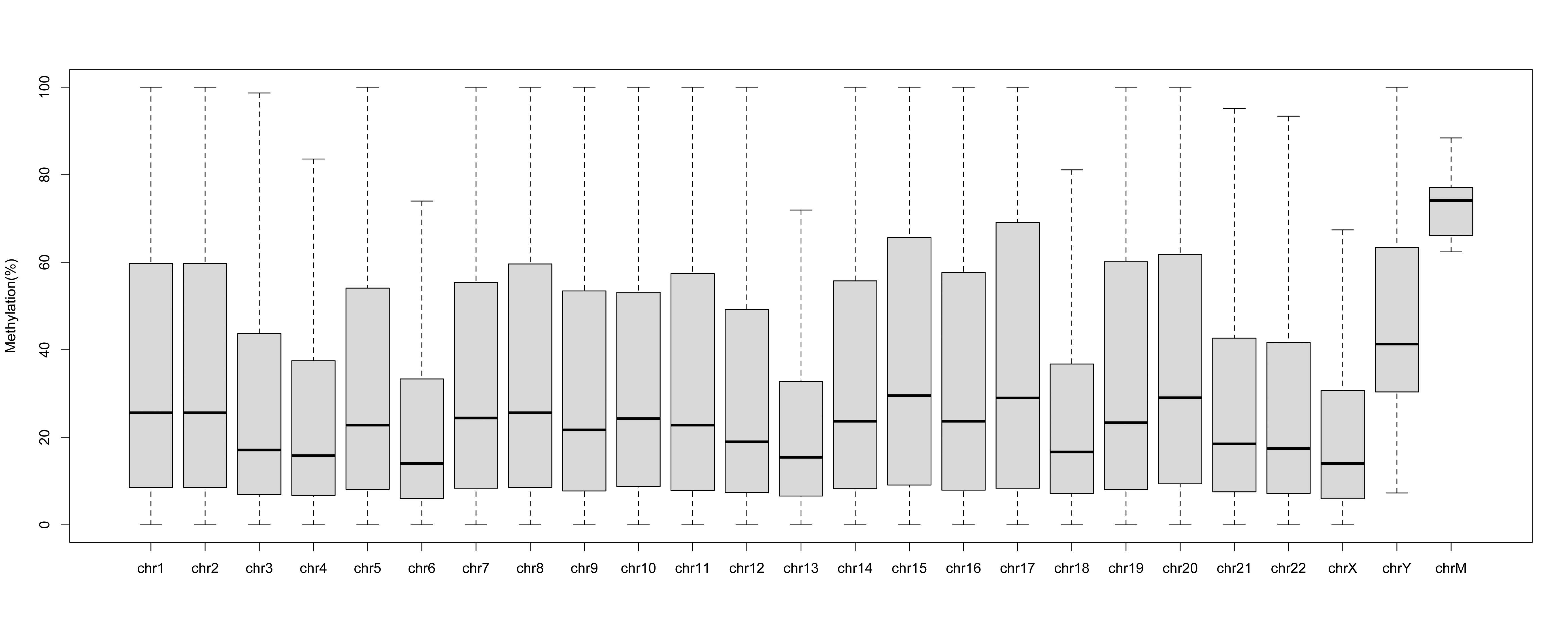
**

**Supplementary Figure S2:** The overall methylation of each chromosome of primary A549 cells. The mitochondrial genome and Y chromosome show significantly high methylation level. The average methylation level of other chromosomesis approximately 20%.

**Supplementary Figure S3**

**
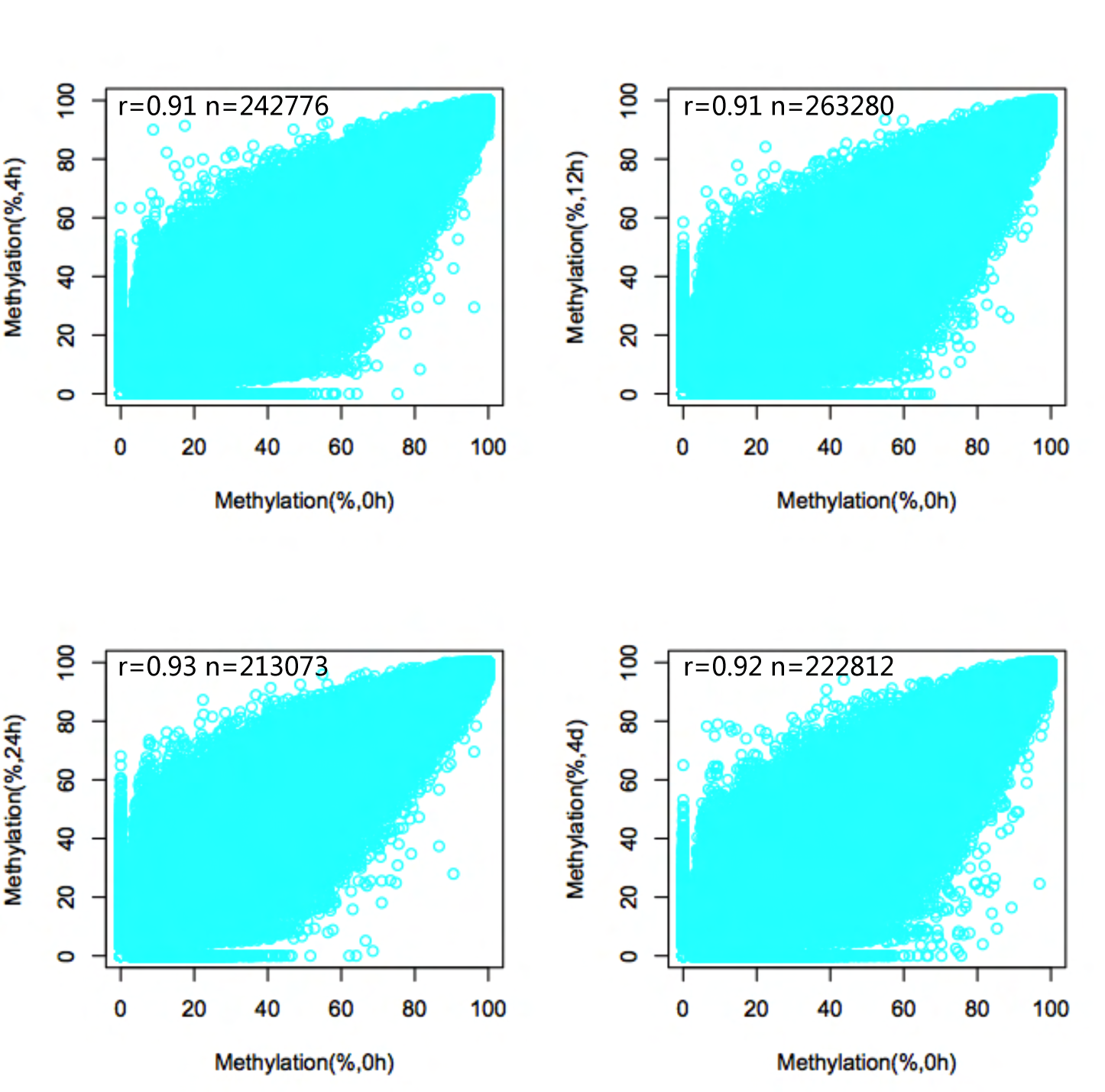
**

**SupplementaryFigure S3:** The methylation level of CCGG sites with 30+ reads in S4h, S12h, S24h and S4d compared with S0h. The Pair-wise correlation coefficients are showed and a similarity between cells before and after EMT is observed.

**Supplementary Table S1:** List of antibodies used for western blot.

| **Antigen** | **Product No.** | **Company** |
| --- | --- | --- |
| E-Cadherin | #4065 | CellSignaling Tech. |
| N-Cadherin | 610920 | BD Transduction |
| Vimentin | #3932 | CellSignaling Tech. |
| Snail | #4719 | CellSignaling Tech. |
| GAPDH(3B3) | M2006 | Abmart |
| Hsp90 | #4877 | CellSignaling Tech. |
| DNMT1 | #5032 | CellSignaling Tech. |
| DNMT3a | SC-10232 | Santa Cruz |
| DNMT3b | SC-70984 | Santa Cruz |
| TET1 | A1506 | AB Clonal |
| TET2 | SC-136926 | Santa Cruz |
| TET3 | SC-139186 | Santa Cruz |
| Histone 3 Trimethyl Lsy36 | NB21-1253 | NOVUS Biologicals |
| Histone 3 Trimethyl Lsy4 | NB21-1023 | NOVUS Biologicals |
| Histone 3 Dimethyl Lsy9 | NB21-1072 | NOVUS Biologicals |

**Supplementary Table S2:** List of primers used for qRT-PCR.

| **Prmer** | **Sequence** |
| --- | --- |
| TET1_forward | CATCAGTCAAGACTTTAAGCCCT |
| TET1_reverse | CGGGTGGTTTAGGTTCTGTTT |
| TET2_forward | GGCTACAAAGCTCCAGAATGG |
| TET2_reverse | AAGAGTGCCACTTGGTGTCTC |
| TET3_forward | TCCAGCAACTCCTAGAACTGAG |
| TET3_reverse | AGGCCGCTTGAATACTGACTG |
| DNMT1_forward | AGGCGGCTCAAAGATTTGGAA |
| DNMT1_reverse | GCAGAAATTCGTGCAAGAGATTC |
| DNMT3a_forward | AGTACGACGACGACGGCTA |
| DNMT3a_reverse | CACACTCCACGCAAAAGCAC |
| DNMT3b_forward | AGGGAAGACTCGATCCTCGTC |
| DNMT3b_reverse | GTGTGTAGCTTAGCAGACTGG |
| TDG_forward | TCACACTCTACCAGGGAAGTATG |
| TDG_reverse | ACGTCCTCCTTCACGAAATTCT |
| E-cadherin F | TGCCCAGAAAATGAAAAAGG |
| E-cadherin R | CTGGGGTATTGGGGGCATC |
| N-cadherin F | TTGGATCAATGTCATAATCAAGTGCTGTA |
| N-cadherin R | CTCCTATGAGTGGAACAGGAACG |
| vimentin F | GACAATGCGTCTCTGGCACGTCTT |
| vimentin R | TCCTCCGCCTCCTGCAGGTTCTT |
| Snail 147F | GCTGCAGGACTCTAATCCAGA |
| Snail 230R | ATCTCCGGAGGTGGGATG |
| β-Actin F | CCTGGCACCCAGCACAAT |
| β-Actin R | GGGCGGGACTCGTCATAC |

**Supplementary Table S3:** List of Index used in second-generation sequencing

| **Library name** | **Index** | **Sequence** |
| --- | --- | --- |
| **S0h-H** | **A7** | CAGATC |
| **S0h-M** | **A8** | ACTTGA |
| **S4h-H** | **A10** | TAGCTT |
| **S4h-M** | **A11** | GGCTAC |
| **S12h-H** | **A1** | ATCACG |
| **S12h-M** | **A2** | CGATGT |
| **S24h-H** | **A4** | TGACCA |
| **S24h-M** | **A5** | ACAGTG |
| **S4d-H** | **A7** | CAGATC |
| **S4d-M** | **A8** | ACTTGA |

**Supplementary Table S4:** Summary of second-generation sequencing data in MSCC-seq.

| **Library name** | **Number of reads with appropriate adaptors** | **Number of mapped reads (percentage)** | **Number of CCGG sites seen at least once** | **Average number of reads per CCGG site** |
| --- | --- | --- | --- | --- |
| **S0h-H** | **33,394,561** | **19,763,515 (59.8%)** | **1,242,492** | **15.9** |
| **S0h-M** | **23,366,511** | **10,444,519 (44.7%)** | **1,312,304** | **8.0** |
| **S4h-H** | **33,912,368** | **19,939,704 (58.8%)** | **1,280,589** | **15.6** |
| **S4h-M** | **17,966,943** | **7,942,158 (44.2%)** | **1,264,960** | **6.3** |
| **S12h-H** | **38,062,401** | **21,738,329 (57.1%)** | **1,316,143** | **16.5** |
| **S12h-M** | **21,024,652** | **9,219,170 (43.8%)** | **1,290,240** | **7.1** |
| **S24h-H** | **32,098,653** | **19,183,274 (59.8%)** | **1,248,699** | **15.4** |
| **S24h-M** | **22,822,806** | **10,629,945 (46.6%)** | **1,335,541** | **8.0** |
| **S4d-H** | **31,243,767** | **18,202,238 (58.3%)** | **1,242,510** | **14.6** |
| **S4d-M** | **24,124,128** | **10,229,623 (42.4%)** | **1,284,331** | **8.0** |

**Supplementary Table S5:** The top 20 genes with the highest and lowest averagemethylation level around TSS region.

| **AccessionNumber**  **Number** | **Gene Symbol**  **Symbol** | **MethylationLevel (%)** | **Gene Expression**  **Expression** |  | **AccessionNumber**  **Number** | **Gene Symbol**  **Symbol** | **Methylation Level (%)**  **Level (%)** | **Gene Expression**  **Expression** |
| --- | --- | --- | --- | --- | --- | --- | --- | --- |
| NM_001018115 | FANCD2 | 0.00 | 2.34 |  | NM_003890 | FCGBP | 91.25 | 3.16 |
| NM_001130997 | FAM58A | 0.00 | 3.11 |  | NM_144691 | CAPN12 | 86.79 | 2.39 |
| NM_004964 | HDAC1 | 0.00 | 4.00 |  | NM_001143785 | FES | 85.57 | 2.38 |
| NM_016039 | C14orf166 | 0.00 | 4.20 |  | NM_012116 | CBLC | 85.25 | 1.86 |
| NM_001243799 | TSC22D1 | 0.78 | 4.26 |  | NM_001199570 | CLIP3 | 84.05 | 2.23 |
| NM_020909 | EPB41L5 | 0.83 | 2.58 |  | NM_021902 | FXYD1 | 83.41 | 2.08 |
| NM_018676 | THSD1 | 1.11 | 0.88 |  | NM_001160418 | ZBP1 | 82.80 | 1.77 |
| NM_033296 | MRFAP1 | 1.13 | 4.22 |  | NM_005270 | GLI2 | 81.67 | 2.51 |
| NM_021647 | MFAP3L | 1.17 | 2.40 |  | NM_130849 | SLC39A4 | 80.06 | 3.21 |
| NM_001142548 | RAD54L | 1.19 | 2.60 |  | NM_003695 | LY6D | 79.98 | 2.31 |
| NM_015646 | RAP1B | 1.23 | 3.94 |  | NM_152286 | PNPLA7 | 79.36 | 2.13 |
| NM_006951 | TAF5 | 1.30 | 2.63 |  | NM_003332 | TYROBP | 78.50 | 2.19 |
| NM_005001 | NDUFA7 | 1.33 | 2.23 |  | NM_032728 | PPAPDC3 | 77.69 | 1.72 |
| NM_145230 | ATP6V0E2 | 1.34 | 3.17 |  | NM_000256 | MYBPC3 | 77.31 | 2.52 |
| NM_002807 | PSMD1 | 1.39 | 3.73 |  | NM_020956 | PRX | 76.60 | 2.47 |
| NM_006886 | ATP5E | 1.41 | 4.35 |  | NM_173664 | ARL10 | 76.47 | 2.40 |
| NM_001009932 | DNASE1L1 | 1.45 | 3.10 |  | NM_194322 | OTOF | 75.34 | 2.12 |
| NM_021199 | SQRDL | 1.46 | 3.90 |  | NM_033046 | RTKN | 74.84 | 2.44 |
| 001135101 | CRELD2 | 1.54 | 3.31 |  | NM_173059 | ZAN | 74.41 | 1.97 |
| NM_032290 | ANKRD32 | 1.61 | 2.31 |  | NM_198478 | NKPD1 | 74.40 | 2.73 |
